# Multiple sequence-directed possibilities provide a pool of nucleosome position choices in different states of activity of a gene

**DOI:** 10.1186/1756-8935-2-4

**Published:** 2009-03-16

**Authors:** Vinesh Vinayachandran, Rama-Haritha Pusarla, Purnima Bhargava

**Affiliations:** 1 Centre for Cellular and Molecular Biology, (Council of Scientific and Industrial Research), Uppal Road, Hyderabad-500007, India

## Abstract

**Background:**

Genome-wide mappings of nucleosome occupancy in different species have shown presence of well-positioned nucleosomes. While the DNA sequences may help decide their locations, the observed positions *in vivo *are end-results of chromatin remodeling, the state of gene activity and binding of the sequence-specific factors to the DNA, all of which influence nucleosome positions. Thus, the observed nucleosome locations *in vivo *do not reflect the true contribution of DNA sequence to the mapped position. Moreover, the naturally occurring nucleosome-positioning sequences are known to guide multiple translational positionings.

**Results:**

We show that yeast *SNR6*, a gene transcribed by RNA polymerase III, constitutes nucleosome-positioning sequence. In the absence of a chromatin remodeler or any factor binding, the gene sequence confers a unique rotational phase to nucleosomes in the gene region, and directs assembly of several translationally positioned nucleosomes on ~1.2 kb DNA from the gene locus, including the short ~250 bp gene region. Mapping of all these gene sequence-directed nucleosome positions revealed that the array of nucleosomes in the gene upstream region occupy the same positions as those observed *in vivo *but the nucleosomes on the gene region can be arranged in three distinct registers. Two of these arrangements differ from each other in the position of only one nucleosome, and match with the nucleosome positions on the gene in repressed and active states *in vivo*, where the gene-specific factor is known to occupy the gene in both the states. The two positions are interchanged by an ATP-dependent chromatin remodeler *in vivo*. The third register represents the positions which block the access of the factor to the gene promoter elements.

**Conclusion:**

On a gene locus, multiple nucleosome positions are directed by a gene sequence to provide a pool of possibilities, out of which the preferred ones are selected by the chromatin remodeler and transcription factor of the gene under different states of activity of the gene.

## Background

Nucleosomes, the fundamental building blocks and repeating units of eukaryotic chromosomes, not only pack the genome but also participate in gene regulation [1]. The position of nucleosomes must be well defined in order to ensure proper control of all DNA-related activities, as folding of DNA by the histones and positioned nucleosomes can help to establish contact between two remotely placed transcriptional factors [2,3]. Genome-wide mapping has shown inverse correlation of nucleosome occupancy with promoter strength and transcription initiation rate [4,5]. Genes show well-defined patterns of positioned nucleosomes with respect to transcription initiation site (+1 site), transcription factor binding sites and the transcribed regions [6]. Yeast promoters show low nucleosome density [7] while the coordination of nucleosome positions with gene activity is a complex process involving interactions between nucleosomes, transcription factors, histone-modifying enzymes and chromatin remodelers [8].

The location of nucleosomes on the DNA can be dictated by trans-acting factors as well as DNA sequences [9-11]. While nucleosomes can get translationally positioned by aligning next to DNA-bound proteins [12-14], cells probably use the sequence preferences of nucleosomes for regulating the binding site accessibility of transcription factors [15-18]. Some naturally occurring positioning sequences are reported to be responsible for positioned nucleosomes on gene regions *in vivo *in the absence of any bound trans-acting factors [19-22]. Genomic DNA with both low and high-affinity sequences for histone octamers could carry a code for nucleosome arrangement guided solely by DNA sequence. In agreement with this, a comparative genomics study has demonstrated that the organization of nucleosome positioning sequences in the yeast genome can be used to predict genome-wide nucleosome positions [23]. Earlier reports had suggested that ~95% of chicken genomic DNA does not show a histone affinity different from synthetic, random DNA sequences [24]. However, higher-resolution data over large contiguous regions of yeast DNA revealed that ~70% of the nucleosomes on chromosome III are well positioned [25]. It was further suggested that ~50% nucleosome positions *in vivo *are encoded by the genomic DNA sequence [26]. More recently, a complete high-resolution map of nucleosome occupancy across the whole genome of yeast has shown that 81% of the yeast genome is covered by positioned nucleosomes [27]. Sequence-directed nucleosome positioning *in vivo *is further regulated in trans by ATP-dependent nucleosome remodeling complexes [28-30]. Histone variant H2A.Z is also shown to regulate gene activity and nucleosome positioning genome wide [6,31-33].

One of the naturally occurring positioning sequences, 5S rDNA, which is reported to give multiple translationally positioned nucleosomes with unique rotational setting [34], belongs to the class I genes transcribed by the RNA polymerase III (pol III). The yeast *SNR6 *gene, which codes for the U6 snRNA, represents class III of the pol III-transcribed genes [35]. The promoter architecture of *SNR6 *shows an unusual combination of an upstream TATA box, intragenic A box and downstream B box [36] to which the basal transcription factors TFIIIB and TFIIIC bind. Previous studies on *SNR6 *in our lab have shown a good correlation between the *in vivo *chromatin structure [37] and factor-dependent chromatin structure *in vitro *[3,38]. The *in vivo *chromatin structure of *SNR6 *is reported to have an array of positioned nucleosomes upstream of the TATA box and downstream of A box, flanking a nucleosome-free region between TATA box and A box in the active state of the gene [37,39]. In a similar genome-wide arrangement of nucleosomes on pol II-transcribed genes in yeast, positions of the two flanking nucleosomes are reportedly specified by the DNA sequence [40]. As the *SNR6 *gene is always occupied by its basal factor TFIIIC *in vivo *[41-43], the contribution of the gene sequence in establishing the chromatin structure is difficult to ascertain *in vivo*. This may be true for many other genes as well, which are persistently occupied by their transcription factors *in vivo*. The salt gradient dialysis method of chromatin assembly *in vitro *deposits nucleosomes in a sequence-dependent fashion and this method has been useful in checking the ability of various DNA sequences to position nucleosomes *in vitro*. Using this method of chromatin assembly, we show here that the *SNR6 *gene sequence has intrinsic nucleosome-positioning signals for discrete translational positions and unique rotational setting, which results in alignment of an array of positioned nucleosomes in both directions on the gene-flanking regions. At any given time, two nucleosome positions on the gene can be contiguous with the array in the 5' upstream region, giving three possible registers. Our results explain how the gene sequence-directed, multiple nucleosome positioning may help establish the chromatin structure of the gene locus *in vivo*.

## Methods

### Plasmids DNAs

Plasmids 601 (pGEM3Z-601) carrying the 601 positioning sequence [44,45] and the plasmids p-539H6 and pCS6 [46] carrying different lengths of yeast genomic DNA harboring the *SNR6 *gene were gifts. All numbers in this study, describing the base pair (bp) positions in the gene are with reference to the transcription initiation site at +1. Plasmid p-539H6 carries the 1180 bp gene region from -539 to +629 bp positions while pCS6 contains the 432 bp DNA from the positions -120 to +312 bp. Plasmids pU6 5'half and pU6 3'half were constructed by inserting the PCR-amplified *SNR6 *gene regions into the vector DNA.

### Chromatin assembly

Chromatin was assembled by the salt gradient dilution method as described earlier [14], except that the core histones were from Drosophila embryos. As sequence-dependent nucleosome positioning is influenced by octamer concentration [47], a histone octamer titration for each DNA was carried out before choosing the DNA:histone ratio for each reconstitution. Reconstituted chromatin samples were subsequently digested with micrococcal nuclease (MNase) or DNaseI to carry out further analysis.

Mononucleosomes were assembled on DNA fragments of more than 200 bp size to avoid the end-positioning effect. DNA fragments were PCR amplified from plasmids using appropriate primer pairs having one of the primers 5'-[^32^P]-end labeled. The PCR products were gel purified and per reaction ~10,000 to 20,000 cpm of labeled probe was mixed with the parent plasmid DNA for chromatin reconstitution by salt dilution method. After the reconstitution, 10 μl samples were loaded on 5% native polyacrylamide gel. Gels were dried after the run and retarded mobility of the reconstitute was ascertained by the Phosphor Imaging (Fuji) to visualize the mononucleosome assembly.

### Chromatin structure analysis

Chromatin was subjected to MNase or DNaseI digestion for the low-resolution indirect end-labeling (IEL) analysis or primer-extension footprinting, respectively, as described earlier [14]. All the plasmids have unique sites for the restriction enzyme *Alw*N1 ~0.8–1.2 kb away from the gene regions in the vector DNA, which was used for the secondary digestion of the MNase-digested naked DNA or chromatin for the IEL analysis. A protection of 145 bp or larger size DNA seen in IEL analyses was taken as the indicative of a positioned nucleosome. Image Gauge software (Fuji) was used to generate the profiles of partially digested and gel-resolved naked DNA and chromatin samples from the phosphorimages of the footprinting gels. All protections were ascertained by matching the profiles of the lanes with similarly digested DNA.

### Hydroxyl radical footprinting

Mononucleosomes were reconstituted over PCR-amplified DNA fragments 5'-[^32^P]-end-labeled on either of the strands. Hydroxyl radical cleavage of the DNA was followed on both the strands in 60 μl reaction volumes. Briefly, 5 μl of 1 mM Fe(II)/2 mM EDTA and 10 μl of 10 mM sodium ascorbate are put together on the sides of the tube, to which 10 μl of 0.6% wt/vol. H_2_O_2 _is added and immediately mixed with the reconstituted chromatin/naked DNA [48]. The cleavage was allowed to proceed for 3 and 5 minutes and the reaction was quenched by the addition of 10 μl of 100 mM thiourea. The DNA sample was cleaned by phenol:chloroform (1:1) extraction, ethanol precipitated and resolved in 8% denaturing urea-acrylamide gel. Gels were dried, exposed for phosphorimaging and profiles were generated using Image Gauge software from Fuji.

### Exonuclease III footprinting

Nucleosome positions were mapped on DNA, 5'-[^32^P]-end-labeled on one of the strands [49]. The reconstituted mononucleosomes were digested with 20 U/ml Exonuclease III (NEB), for 0, 3, 6, and 9 minutes as compared with 0, .5, 1.5, and 2 minutes for the naked DNA samples. The digestion was stopped by adding the 10× exonuclease stop buffer having 0.5 mg/ml proteinase K, 200 mM Tris-HCl pH8, 50 mM EDTA and 5%SDS. DNA was phenol extracted, ethanol precipitated and resolved on the 8% urea-acrylamide denaturing gel. Gels were dried after the run and exposed to the Phosphor Imaging screen (Fuji). Bands appearing first and remaining resistant to ExoIII digestion during the time course were taken as nucleosome boundaries.

## Results

**Figure 1:**
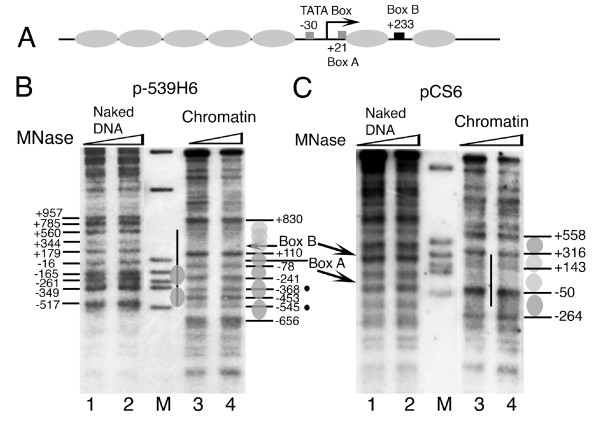
**Sequence-directed nucleosome positions on the yeast *SNR6 *gene**. Numbers denote the positions of the promoter elements and MNase cuts in the genomic DNA in base pairs while ovals represent individual nucleosomes. (A) Schematic representation of reported nucleosome positions *in vivo *on the gene and its flanking regions. Arrow marks the transcription initiation site. (B) and (C) Indirect end-labeling analysis of the chromatin structure reconstituted on the *SNR6 *gene in the plasmids *in vitro*. Naked DNA (lanes 1, 2) and chromatin (lanes 3,4) were digested with MNase and probed with a primer away from the gene region. Positions of the boxes A and B are marked, M denotes molecular size marker and the vertical bar marks the genomic DNA region in the plasmid. (B) Primer hybridizes 1281 bp upstream of the *SNR6 *TATA box. Bands at -545, -656 and +830 bp map in the vector DNA. As compared with -241 and -453, bands at -368 and -545 are faint (black dots, lanes 3 and 4) and may not be the chromatin-specific cuts (cf. naked DNA cuts at -349 and -517, lanes 1 and 2). Two alternate registers are shown on two sides of lanes 3 and 4. (C) Primer hybridizes 894 bp upstream of the TATA box. Positions -264 and +558 fall in the vector DNA.

As shown in Figure 1A, the *SNR6 *gene locus constitutes a TATA box at -30 bp, box A at +21 bp, the terminator at +109 bp, and box B at +233 bp positions (with respect to +1 at transcription initiation site). To find the contribution of the genomic DNA sequence to the nucleosome positions, we used the salt gradient dilution method to deposit nucleosomes on plasmids carrying different parts of genomic DNA from the *SNR6 *gene region in the absence of any bound transcription factor, and subjected the chromatin to structural analyses for locating the positioned nucleosomes, if any.

### Yeast SNR6 locus is covered by positioned nucleosomes

The plasmid p-539H6 carrying ~1.2 kbp of the genomic DNA from the *SNR6 *locus (marked with a vertical line, Figure 1B) shows significant differences between MNase digestion patterns of the chromatin and naked DNA in the IEL assay. The region downstream of the gene terminator at +110 bp shows ~700 bp-long protected region, suggesting this region may be covered by rotationally phased nucleosomes, probably with overlapping translational positions. Digestion of the ~540 bp-long naked and chromatin DNA upstream of +110 bp by MNase shows significant differences. Mapping of the MNase-cut positions revealed the presence of a translationally positioned nucleosome between the +110 and -78 bp and an array of nucleosomal-size protections (marked with gray ovals) upstream of it, which can be arranged in two distinct registers. Those marked on the right-hand side of lanes 3 and 4 may have three positions -78 to -241, -241 to -453 and -453 to -656 bp in one register (positions 3–5, Table 1) while those marked on the left-hand side at -78 to -368 and -368 to -545 bp (positions 1 and 2, Table 1) may be in another register. A deletion of 2 bp in box B *in vivo*, which abolishes TFIIIC binding, was reported to result in rearrangement or destabilization of the nucleosomes in the gene-flanking regions [39], suggesting these nucleosomes *in vivo *are organized by TFIIIC-dependent chromatin remodeling. It is interesting to note that the *in vivo *structure was reported to have boundaries of two upstream positioned nucleosomes at base pairs -537 and -367 [39]. Thus, it is possible that nucleosome positions 1 and 2 (Table 1) represent the possible nucleosome locations in the presence of TFIIIC, while nucleosome 5 represents a position in the absence of TFIIIC. The upstream nucleosomal array on the genomic DNA in p-539H6 is similar to that on the *SNR6 *locus *in vivo *[37,39] but structure downstream of +110 bp on the plasmid is different, probably because the gene is occupied by TFIIIC *in vivo *and the chromatin in the gene region is remodeled after TFIIIC binding [37,38].

**Table 1:** Comparison of nucleosome positions on *SNR6*, mapped in this study with those reported in other studies

No.	Position (bps) This study	Similar to position (bp)	Comments	Ref. No.
1.	-545 to -368	-537 to -367	*In vivo*, in the presence of bound TFIIIC	39

2.	-368 to -78	-367 to -214	*In vivo*, in the presence of bound TFIIIC	39

3.	-656 to -453		*In vitro*, sequence-directed	

4.	-453 to -241		*In vitro*, sequence-directed	

5.	-241 to -78	-240 to -70	*In vivo*, in the presence of bound TFIIIC	37

6.	-125 to +25	-194 to -16	*In vivo*, in the absence of bound TFIIIC	39
		
		-120 to +28	*In vivo*, under repression	37

7.	-110 to +45	-116 to +40	Predicted	26

8.	-90 to +65	Positions 8–10 together as bp -78 to + 110 in the plasmid		
			
9.	-60 to +95	p-539H6, Figure 1B, sequence-directed.		
			
10.	-40 to +115	Positions 9–15 together as bp -50 to + 316 in the plasmid		
			
11.	-20 to +125	pCS6, Figure 1C, sequence-directed.		

12.	+51 to +196	+48 to +192	*In vitro*, TFIIIC-dependent	3
		
		+94 to +198	*In vivo*, in the presence of bound TFIIIC	59

13.	+71 to +216	+71 to +227	Predicted	26
		
		+85 to +206	*In vivo*, in the absence of bound TFIIIC	39

14.	+91 to +236		*In vitro*, sequence-directed	

15.	+101 to +256		*In vitro*, sequence-directed	

All 15 positions in Column 2 are sequence-directed as nucleosomes are assembled in the absence of any transcription or chromatin assembly factors. Positions 1 to 5 are mapped by the low-resolution technique (Figure 1B) and fall in the upstream region; the rest of the positions on the gene region (6 to 15) are mapped by the higher resolution Exo III analysis (Figures 5 and 6) in this study. Positions 3 to 5, 8, 9, 14 and 15 represent the register R1 while R2 is constituted by the positions 1, 2 (or 3, 4), 6, 12 and 13. Arrangement of nucleosomes in active state R3 is generated by the positions 1, 2 (or 3, 4), 5 and 12 giving a nucleosome-free region flanked by positioned nucleosomes.

### Box B is buried in the nucleosomal region

In order to know further details of the chromatin structure close to the transcribed region of the gene, we assembled the chromatin *in vitro *on the plasmid pCS6 which carries ~400 bp genomic DNA region (vertical line, from the positions -120 to +312; Figure 1C) from the *SNR6 *gene locus. Southern probing of the MNase digest of the chromatin by the gene-specific and vector DNA-specific probes showed a better nucleosomal ladder on the gene region (data not shown), suggesting the gene has higher affinity for histones. IEL analysis of the MNase digestion patterns of the chromatin and naked DNA control shows that the complete gene region from -50 to +316 bp is protected in chromatin (lanes 3 and 4, Figure 1C). However, the MNase footprinting analysis of this chromatin did not show any boundaries separated by 145 bp nucleosomal-size protections (data not shown). Two nucleosomal-size protections are seen in flanking regions of the gene on the vector DNA also (dark ovals, Figure 1C). Since the positioned nucleosomes are seen only on and around the gene region, compared with the vector DNA, it is possible that the positioning is related to the gene sequence. The MNase digestion patterns and the mapped MNase cut sites on the chromatin assembled over the plasmids p-539H6 and pCS6 do not match, probably due to the sizes of the mapped regions, therefore resolution differences of the gels. Deletion of the DNA upstream of position -140 bp *in vivo *was reported to result in loss of the upstream array of the nucleosomes [39]. Therefore, the absence of the array may be because of the absence of DNA upstream of -120 bp in pCS6. However, the presence of a positioned nucleosome on the gene-flanking vector DNA suggests that the gene sequence directs positioned nucleosomes in its immediate vicinity as well. The absence of a unique translational positioning of nucleosomes and the protection size of 366 bp (-50 to +316 bp), which has box B of the gene in its center, suggest that the whole gene region may be covered with the nucleosomes having unique rotational phasing.

### SNR6 confers a unique rotational phase to nucleosome

**Figure 2:**
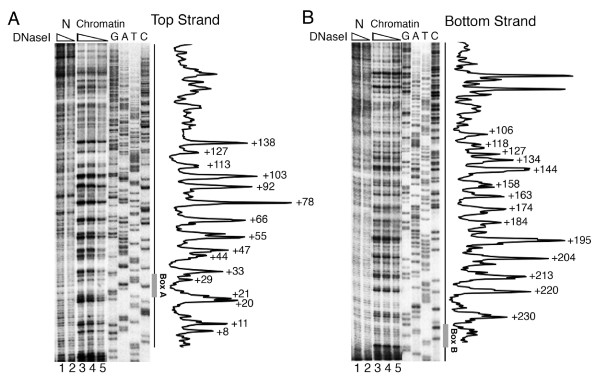
**Chromatin DNA between the boxes A and B has a unique rotational phase**. Gels and profiles from DNase I footprinting analysis of chromatin assembled onto plasmid pCS6 are shown for both the DNA strands. (A) Primer extension probe was located 50 bp upstream of transcription start site, complementary to the *SNR6 *gene bottom strand. Numbers marking the profile in the right panel identify the DNase I cut positions on the top strand. (B) The 5' end of the primer extension probe was 31 bp away from the 3' end of box B, complementary to the *SNR6 *gene top strand. Numbers marking the profile in the right panel identify the DNase I cut positions on the bottom strand.

**Figure 3:**
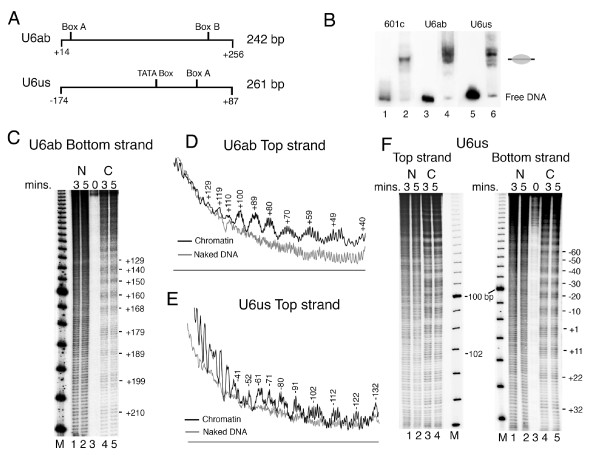
**Nucleosomes on the *SNR6 *gene are rotationally positioned**. (A) Schematic representation of the selected gene regions, PCR-amplified from p-539H6 with their names and sizes is given. (B) Gel mobility shift assay of chromatin assembled over end-labeled 601c, *SNR6*ab and *SNR6*us DNA fragments (lanes 2, 4 and 6, respectively). Lanes 1, 3 and 5 show free DNA controls. Gray oval on the left-hand side marks the position of the centrally positioned nucleosome. (C) to (F) Hydroxyl radical cleavage of the DNA. Numbers identify the cleavage peaks of chromatin in base pairs. (C) and (F) Digestion times are given in minutes; 0 min. represents undigested DNA. Cleavage pattern of the chromatin samples, C, are shown in the lanes 3 and 4 for the top strand gel and lanes 4 and 5 for bottom strand gels. Lanes 1 and 2 show digestion pattern of naked DNA (N), while a 10 bp DNA ladder (Invitrogen) was end-labeled and used as size marker in the lanes M. (D) Profile of the digestion pattern of the top strand of *SNR6*ab chromatin. (E) Profile of the digestion pattern of the top strand of *SNR6*us chromatin from the gel shown in panel (F).

We used DNase I footprinting (Figure 2) and hydroxyl radical cleavage (Figure 3) to find the presence of a 10 bp ladder, characteristic of rotationally positioned nucleosomes, on the gene region. DNase I footprinting of both the strands of the chromatin assembled on the plasmid pCS6 (Figure 2, panels A and B) shows a frequency of cut with ~8 to 12 bp periodicity. DNase I cuts two strands of DNA with a 4 bp stagger and there may be an error of 1 to 2 bp in upper parts of the gel in identifying the cut positions. Nevertheless, combining the mapping on both the strands, a 10 bp periodicity of cuts in the whole region can be seen, suggesting that ~180 bp DNA sequence between the boxes A and B (from +33 bp to +213 bp) may have a rotational nucleosome positioning signal.

Hydroxyl radical cleavage of *SNR6 *chromatin was performed in two parts, on both the strands of each part. U6ab and U6us (Figure 3A) were PCR amplified from the plasmid p-539H6, and mononucleosome assembly on them was monitored by gel shift assay (Figure 3B). Similar to the 601c DNA (lane 2), which gives a centrally positioned nucleosome [50], both U6ab (lane 4) and U6us (lane 6) DNAs show major population of a centrally positioned nucleosome. Additionally, one band for U6ab and three minor bands for U6us can also be seen. Mapping of hydroxyl radical cleavages on both the strands of U6ab DNA, which covers the *SNR6 *gene region from +14 to + 256 bp (Figures 3C and 3D) shows a continuum of 10 bp periodicity on the whole region. Similar mapping on both the strands of U6us carrying the upstream region of the *SNR6 *gene shows a well-pronounced helical periodicity up to -100 bp which appears to be less defined in the region further upstream. This is better revealed by the top strand cleavage pattern (gel in Figure 3F and profile in the Figure 3E). When cleavage maps of both parts are taken together, a periodicity of 10 ± 1 bp is found to prevail in the same phase on the whole gene region from -100 bp to +210 bp. The deviation by 1 bp may be because of the change in periodicity at the dyad axis of a nucleosome [51]. The rotational information of a DNA can influence nucleosome positions [52,53]. Thus, the unique rotational phase of the nucleosomes on the *SNR6 *gene may influence the positions on the flanking genomic DNA regions, as suggested by the results in Figure 1.

### Yeast SNR6 sequence shows two types of nucleosome positions

**Figure 4:**
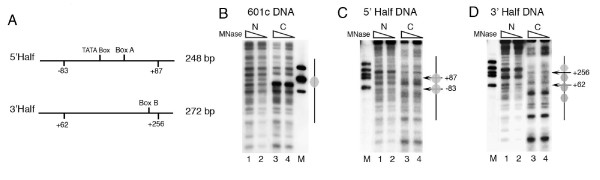
**Nucleosome positioning properties of two *SNR6 *halves are different**. (A) Schematic representation of the selected gene regions PCR-amplified from pCS6 and cloned into a plasmid along with their names and sizes is given. (B), (C) and (D) Indirect end-labeling analysis of the chromatin assembled over the plasmids 601c, 5' half and 3' half respectively. Arrows mark the positions of the genomic DNA ends while ovals mark the positioned nucleosomes. Lanes 1 and 2 show digestion pattern of naked DNA N, while digestion pattern of the chromatin samples C are shown in the lanes 3 and 4 in each panel. Probe was same as that in the Figure 1C while lane M shows molecular size markers.

The complete *SNR6 *gene region from TATA box at -30 to box B up to +242 bp can probably be occupied by two contiguous nucleosomes (Figure 1C). The nucleosome positioning signal on the sea urchin 5S rRNA gene is centered around the +1 position, giving a nucleosome positioned from -78 to +78 bp [54]. As box B of *SNR6 *is further downstream, similar positioning on *SNR6 *would allow formation of one more nucleosome on the gene region, downstream of +90 bp position. Therefore, we separated the *SNR6 *gene sequence into two halves and cloned the bp regions -87 to +83 (5' half of the gene) as well as +62 to +256 (3' half of the gene) into two different plasmids (Figure 4A). We used the IEL technique to further confirm the nucleosome-positioning properties of both the halves in the context of the flanking plasmid vector sequences.

The IEL technique can map nucleosome positions with a fairly good accuracy [55]. For example, IEL of the 601c chromatin shows the presence of a single positioned nucleosome on the synthetic sequence (lanes 3, 4, Figure 4B). Two nucleosomal-size protections (gray ovals) are seen on two sides of the center (+2 bp) of the genomic DNA insert in the 5'-half plasmid (lanes 3, 4, Figure 4C). The positions of the two protections suggest that both of them include the vector DNA and that the DNA on both the sides of the +1 site of *SNR6 *has nucleosome positioning properties. Asymmetry between two halves of a nucleosome is reported to facilitate translational positioning [56,57]. Each half of the insert DNA is less than a nucleosomal size and its continuity with the vector DNA on both sides of the +1 bp position results in its incorporation into the translationally positioned nucleosomes on both sides. This suggests that when present as part of the whole gene, the 5' half may help position nucleosomes on flanking DNA. On the plasmid bearing the 3' half DNA, a positioned nucleosome on the gene region is found flanked by the positioned nucleosomes on both sides (lanes 3, 4, Figure 4D). This suggests that central positioning of a nucleosome on this region (data not shown) leads to the alignment of more nucleosomes on both sides, giving four translationally positioned nucleosomes in the same register. Though both the *SNR6 *halves carry at least 17 helical turns of the DNA with same rotational phase, the 3'-half DNA has probably both rotational and translational positioning signals while the 5' half of *SNR6 *has the signal for unique rotational positioning, in accordance to the nucleosome positionings seen in Figure 3B. Thus, it is possible that both the halves of the gene retain their positioning properties in isolation, even when flanked by other non-genomic DNA sequences.

### Rotational positioning of the nucleosomes on the 5' half of the gene

**Figure 5:**
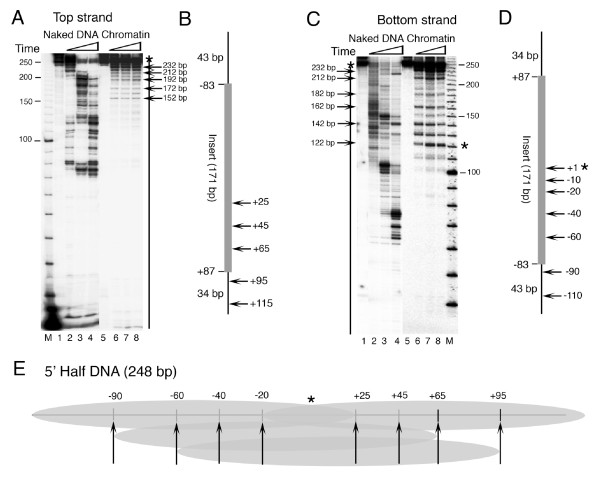
**Exo III mapping of the nucleosomes on the 5' Half *SNR6 *DNA fragments**. Chromatin was assembled on the 248 bp DNA fragments labeled at the 5' end of either the top strand (panel A) or the bottom strand (panel B). A 10 bp DNA ladder (Invitrogen) was end-labeled and used as size marker in the lanes M. Sizes of the marker bands are given in the left-hand side of both the panels while arrows on the right-hand side give the sizes of the DNA fragments due to the pauses of Exo III. Lanes 1 and 5 in both the panels show the uncut DNA. Other lanes show naked DNA or chromatin digested for different times by Exo III. (C) Schematic representation of mapping results from the panels A and B. Insert represents the cloned genomic DNA from +62 to +256 bp positions in the *SNR6 *gene while the flanking 48 and 34 bp are vector-derived DNA. The numbers on right-hand side are the Exo III pauses seen in the gels and represent the nucleosome boundaries.

To dissect the gene sequence further with respect to its nucleosome positioning capability, we reconstituted mononucleosomes over DNA fragments of ~240 to 270 bp sizes (Figure 4A) carrying the two halves of the gene and mapped the locations of nucleosomes on them by using exonuclease III (Exo III) digestion assay. As compared with the IEL method, which does not differentiate between different rotational positions, Exo III mapping of nucleosomes positioned on the 5'-half DNA (Figure 5) shows the presence of several translational positions with a unique rotational phase. Mapping of the Exo III-resistant positions on both the strands, which are not well resolved in the top of the gel (lanes 6–8, panel A), shows the presence of multiple positions with 10 bp differences (lanes 6–8, panels A-D). Exo III shows a prominent pause at the +1 (marked by asterisk, 122 bp band) and -110 (232 bp band) base pair positions on the bottom strand of *SNR6 *(panels C and D). Out of several possibilities, mapping of both the boundaries of at least four major positions (Table 1, position nos. 7 to 10), reveals the nucleosome boundaries are 155 bp apart. Taking 156 bp as the nucleosomal size, a sequence-based nucleosome position from -116 to +40 bp, similar to position number 7 (Table 1) has been predicted on the *SNR6 *DNA ([26], ). Application of 145 bp nucleosome size to the most interior Exo III pauses at -20 bp on the bottom strand and +25 bp on the top strand (a comparatively weaker pause at 152 bp size DNA in lanes 6–8, panel A) would place the ends of the two nucleosomes at +125 and -120 bp, respectively, (positions 11 and 6, Table 1) at the extreme ends of this DNA (panel E). Thus, -20 to +25 bp of *SNR6 *represents a DNA stretch, central and common in all possible nucleosome positions on the 5' half of *SNR6 *(panel E), which brings the +2 bp of *SNR6 *to the center of all the possible positions. Exo III shows a pause at the +1 bp position (panels D and E, the asterisk), which shows alignment of two nucleosomes on its two sides in the 5'-half DNA (Figure 4B), suggesting this may be a central, reference position for organization of nucleosomes on this helically phased DNA. These results suggest that depending on the context, nucleosomes either exclude or assemble over the +1 site. The possibility of multiple positions at uniform intervals on the 5'-half DNA suggests that the protection seen upstream of +110 bp position in the plasmid p-539H6 (Figure 1B) may be due to the translationally positioned nucleosomes with unique rotational settings and not due to a unique translational position. Thus, a similar positioning may be observed *in vivo *due to this sequence, present as part of the full-length gene.

### Translationally positioned nucleosomes on 3' half of the gene

**Figure 6:**
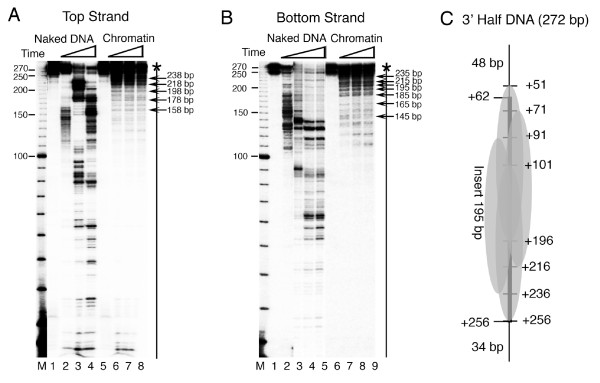
**Exo III mapping of the nucleosomes on the 3' Half *SNR6 *DNA fragments**. Chromatin was assembled on the 248 bp DNA fragments labeled at the 5' end of either of the strands and digested by Exo III for different times. A 10 bp DNA ladder (Invitrogen) was end-labeled and used as size marker in the lanes M. (A) Mapping on the top strand. Lanes 1 and 5 show uncut DNA; lanes 2 to 4 show naked DNA and 6 to 8 show chromatin. Sizes of the marker bands are given on the left-hand side while arrows on the right-hand side give the sizes of the DNA fragments due to the pauses of Exo III. (B) Schematic summary of mapping results from the panel A. Insert represents the cloned genomic DNA from -83 to +87 bp positions in the *SNR6 *gene while the flanking 43 and 34 bp are vector-derived DNA. The numbers on the right-hand side are the Exo III stops seen in the gel and represent the nucleosome boundaries. (C) Mapping on the bottom strand. Lanes 1 and 5 show uncut DNA; lanes 2 to 4 show naked DNA and 6 to 8 show chromatin. Sizes of the marker bands are given in the right-hand side while arrows on the left-hand side give the sizes of the DNA fragments due to the pauses of Exo III. (D) Schematic summary, similar to panel B, of the mapping results from the panel C. The numbers on right-hand side are the Exo III stops seen in the gels and represent the nucleosome boundaries. (E) Schematic representation of mapping results on 5' half DNA from both the strands. Ovals represent nucleosomes while arrows show positions of their boundaries and Exo III stops, marked in upper portion of the cartoon. An asterisk in panels C, D and E shows the position of Exo III stop in the middle of the DNA fragment, at +2 bp position of *SNR6*.

Exo III mapping of the centrally placed nucleosome on the 3'-half DNA (Figure 6) shows clustering of strong Exo III pauses at equal distances from the 3' end of both the strands, suggesting symmetrical and central placements of the nucleosomes with strong boundaries. Strong pauses of Exo III with 10 bp frequency is observed on both the strands. At least, 54 to 57 bp DNA is digested by Exo III from both the ends (218 and 215 bp-size products, panels A and B). Panel B shows a series of strong Exo III-resistant boundaries with 10 bp differences digesting up to 87 bp (185 bp-size product in gel) from the 3' end of the bottom strand. Mapping of the Exo III-resistant positions on both the strands shows nucleosomal boundaries separated by 145 bp. Thus, taking 145 bp as the nucleosomal size, four translational positions (gray ovals, Figure 6C) with unique rotational phase can be deduced from mapping on both the strands of this DNA (positions 12 to 15, Table 1). The position +71 to +216 may be the same as the position +71 to +227 predicted by the DNA sequence, which takes 156 bp as the nucleosomal size ([26], ). Thus, the 3' half of the *SNR6 *gene may have strong signals for both translational and rotational nucleosome positionings.

### Sequence-directed nucleosomal arrangement on the SNR6 gene

Nucleosome positioning on the mouse mammary tumor virus long terminal repeat (MMTV-LTR) is suggested to result from the additive effects of multiple sequence-related features of the DNA [58]. Taken together with this conclusion, the current study on the nucleosome positionings on and around the *SNR6 *gene shows that the *SNR6 *gene sequence constitutes strong rotational and translational positioning signals, which may influence each other in defining several alternative nucleosome positions on the gene. Centrally placed nucleosomes on the 3' half of the gene have four possible translational positions whereas several rotationally phased nucleosomes on the 5' half are more concentrated towards its 5' end. Sequences around the +1 site and between the boxes A and B of the gene play an important role in organizing the chromatin structure of the gene.

**Figure 7:**
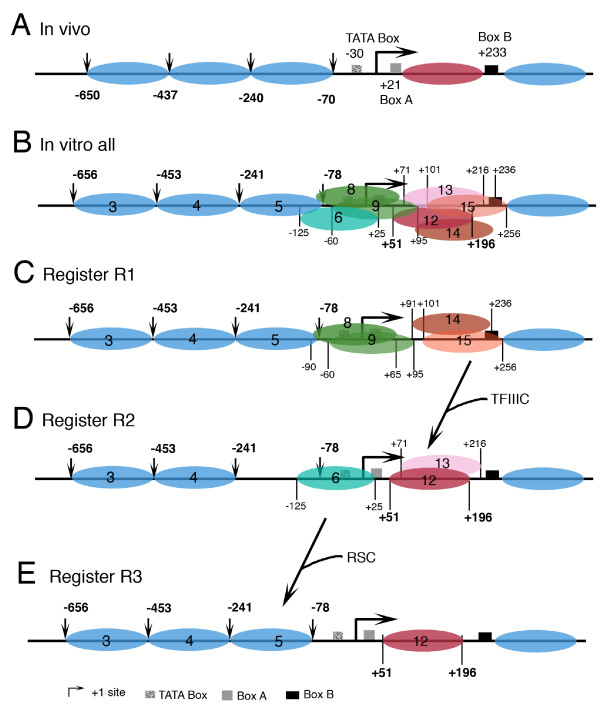
**Comparison of the nucleosome positions on the *SNR6 *gene locus**. TFIIIC is omitted for the sake of clarity. Positions of the promoter elements are marked in panel A and described at the bottom of the figure. Numbers in bold and vertical arrows represent the MNase cut sites mapped by the indirect end-labeling technique in the upstream region. The rest of the numbers in panel B mark the positions of nucleosome boundaries mapped by Exo III footprinting. Nucleosomes are color coded, with their positions as given in Table 1. (A) Positions reported *in vivo *in the presence of TFIIIC [37]. (B) All the *in vitro *positions in the absence of TFIIIC, as mapped in this study and summarized in Table 1. Positions 1, 2 and 7 are omitted for the sake of clarity. (C, D and E) show three possible registers (R1, R2 and R3) generated by the combinations of positions depicted in the panel (B) under three different conditions. Positions 5 and 6 are mutually exclusive. *In vivo*, 6 is occupied in repressed state (Panel D), while chromatin remodeler RSC shifts it to position 5 in active state (Panel E). Boundaries of position 12, which is selected after TFIIIC binding and chromatin remodeling *in vivo *are marked in bold.

Most of the positions mapped in this study and summarized in Table 1 can be correlated to the positions reported earlier [3,37,39,59] or predicted by the *SNR6 *sequence [26]. Figure 7 compares a summary of the results of this study (*in vitro*, panel B) with the nucleosome positions from our previous study ([37], *in vivo*, panel A). The striking similarity of the mapped positions in the gene upstream region (in bold, panel A) and less defined rotational phase of the DNA upstream of -100 bp (Figure 3) suggests that the nucleosomes upstream of -70 bp position are directed by the *SNR6 *sequence. With a strong possibility of multiple sequence-directed positions at uniform intervals on the *SNR6 *gene region (Figures 4, 5, 6), when both the halves of the *SNR6 *are together in the gene, the nucleosome positions on the 5' half of the gene may align with those in the downstream region.

Two different registers (R1 and R2, Figure 7C and 7D) of the nucleosome positions on the gene region may be possible under different conditions, along with alignment of positioned nucleosomes on both the gene flanking regions. In the register R1, the 5' nucleosome may be centered around the +1 site while downstream nucleosome may be more towards the 3' end of the gene, covering the box B. This arrangement could be possible by selecting either the nucleosome positions 8 and 14 or 9 and 15 to align with the position 5 in the upstream region (Figure 7C). While nucleosomes 14 and 15 would cover the box B at their 3' ends, nucleosomes 8 and 9 would block the TATA box to A box region. In the second alternative (R2), nucleosome 6 may align with the nucleosome 12 or 13 and generate a condition in which TFIIIC can occupy boxes B and A but TATA box and +1 site remain masked. However, the presence of nucleosome 6 would exclude the possibility of occupancy on the position 5 (Figure 7D). Such an arrangement has been observed *in vivo *under repression when TFIIIC is seen occupying the gene [37,41-43]. Therefore, R1 represents the arrangement in the absence of TFIIIC, while the first step of chromatin remodeling associated with TFIIIC binding [3] may lead to the arrangement R2 (Figure 7D) *in vivo*. While the sequence ensures the gene is covered by positioned nucleosomes, the nucleosomes subsequently occupy one of the sequence-directed positions in the active state to give another arrangement R3 *in vivo*, as a result of the second step of sequential remodeling [38], as discussed below.

## Discussion

### The SNR6 gene has nucleosome positioning signals

Several synthetic and some naturally occurring sequences have been used for the deposition of positioned nucleosomes *in vitro *[56,60,61] but none of them give unique positioning *in vivo*. *SNR6*, similarly, does not have a signal for unique translational positioning. Rotational positioning is determined by the bendability of the involved sequence [62,63]. Therefore, the nucleosome positioning capability of *SNR6 *DNA may be inherent in its DNA sequence, which shows a unique rotational setting. Sequences that have intrinsic curvature, or that are flexible, prefer to be incorporated into nucleosomes since it takes less energy to bend this type of DNA segment around the core histones [45,57]. These sequences do not show any consensus except that the trajectory of the DNA may be bent [45]. We used a simple program, BEND [64], which calculates the magnitude of local bending and macroscopic curvature at each point along a DNA sequence. Output of the program (data not shown) for the *SNR6 *sequence in pCS6 revealed that the *SNR6 *DNA has intrinsically bent DNA segments, suggesting the bending and curvature of this DNA may have a role in the nucleosome positioning. It will be interesting to explore the possibility of using different regions of the naturally occurring *SNR6 *gene sequence for nucleosome positioning *in vitro *and *in vivo*.

### Establishment of SNR6 chromatin structure *in vivo*

Results from this study have shown that in the absence of any factor, several positioned nucleosomes cover the entire gene region. However, all the mapped positions cannot be occupied simultaneously and may be mutually exclusive. Alignment of contiguous translational positions in the ground state would result in nucleosomes completely covering the gene and blocking the access of its factors to all the target sites. Different translational possibilities of the nucleosomes downstream of -140 bp probably represent the sequence-directed positions adopted by nucleosomes under different states of the activity of the gene.

We had shown earlier that TFIIIC can access the box B of *SNR6 *buried in nucleosomes *in vitro *[3], and a nucleosome between the boxes A and B shifts by ~40 bases due to the subsequent chromatin remodeling [38]. As a result, the nucleosome takes a unique translational position between bp +50 and +190 [3]. Thus, the nucleosomes on positions 13 to 15 (Table 1) in the absence of TFIIIC possibly acquire position 12 (Table 1) due to chromatin remodeling after TFIIIC binding *in vivo*. In agreement with this, the sequence +101 to +196, common in the four overlapping positions on the 3' half (Figure 6C), matches well with the reported protection (from +94 to +198 bp) on *SNR6 in vivo *as estimated by MNase footprinting [59]. When TFIIIC binds to the box B, the arrangement R1 is converted to R2, whereby the upstream nucleosome positions 8 and 9 (arrangement R1), covering the box A in the ground state of the gene, would realign and move to the position 6, as a result of TFIIIC-dependent chromatin remodeling *in vivo*. As the nucleosome 6 covers the +1 site and the TATA box, and gives a partial block of the box A at its 3' end, another remodeling will be required to activate the gene. We had reported earlier a sequential remodeling of the *SNR6 *chromatin which shifts the nucleosome from the TATA box to the region -70 to -240 bp (position 5) further upstream [37,38]. Activation of the gene brings the chromatin remodeler RSC [37], which facilitates the upward shift of the nucleosome from the position 6 to position 5, generating the arrangement R3 (Figure 7E), wherein a nucleosome-free region is flanked by two positioned nucleosomes (positions 5 and 12) as found *in vivo *(Figure 7A). The chromatin structure R3, finally generated by these sequence-dependent rearrangements, depends on the remodeler, which leaves the gene in a repressed state [37]. Significantly, similar to the difference between the repressed and active state chromatin structure of the gene [37], R2 and R3 differ from each other only in the position of one nucleosome (position numbers 6 or 5, Table 1, Figures 7D and 7E).

### Sequence-directed positioning of nucleosomes *in vivo*

This study shows that the final nucleosome positions on *SNR6 in vitro *and *in vivo *are influenced by the combined effects of different segments of the gene sequence. As a chromatin remodeler is recruited to the target genes by its factors, the binding of a factor may decide the nucleosome positions in the active and repressed state of a gene region [14]. Several genome-wide studies on nucleosome arrangements have recently suggested that the genome codes its own packaging by having most of the nucleosomes positioned in a sequence-directed manner [23,26]. Different sequence-directed positions of nucleosomes are chosen by different chromatin remodelers as end-products of their remodeling activities. In the absence of the remodeler Isw2 in yeast, nucleosomes were found to adopt sequence-directed positions genome wide [28,29], suggesting a chromatin remodeling is used to choose between the sequence-directed alternate positions of nucleosomes. However, a nucleosome positioning sequence database NPRD [26] has reports on only four genes, which show sequence-directed nucleosomes *in vivo*. On one well-studied yeast gene locus, PHO5, intrinsic properties of the promoter DNA were found to give nucleosome positions similar to those *in vivo *[65]. Similarly, sequence is suggested to play an important role in positioning nucleosomes on yeast CUP1 locus [20] and MMTV 3' LTR DNA [17]. Our studies on *SNR6 *chromatin structure (this study, [3,38]*in vitro *and [37]*in vivo*) establish a strong correlation between the sequence-directed positions of the nucleosomes before and after TFIIIC binding as well as chromatin remodeling. The combined results of these studies show that transcription factor binding and chromatin remodeling modulate the nucleosomal organization of the *SNR6 *gene region *in vivo *when the resultant nucleosome positions are not randomly generated. They are rather chosen from few sequence-directed select possibilities.

## Conclusion

Our results have shown that all the nucleosomes found associated with a gene locus *in vivo *under different states of its activity correspond to one of the multiple positions directed by a genomic DNA sequence. This may be the reason that in contrast to synthetic sequences, which could be designed to give unique positionings, the naturally occurring nucleosome-positioning signals give multiple alternatives and cannot be defined by consensus sequence elements.

## Abbreviations

bp: Base Pair; Exo III: Exonuclease III; IEL: Indirect End-labeling; MMTV-LTR: Mouse Mammary Tumor Virus Long Terminal Repeat; MNase: Micrococcal Nuclease; pol: Polymerase; *SNR6*: U6 snRNA.

## Competing interests

The authors declare that they have no competing interests.

## Authors' contributions

PRH initiated the study, and standardized chromatin assembly and structure analysis protocols. VV carried the study further, constructed plasmid DNAs and carried out footprinting analyses. PB conceived of the study, participated in design and coordination and wrote the manuscript. All authors have read and approved the final manuscript.
