# Correction: Multiple sequence-directed possibilities provide a pool of nucleosome position choices in different states of activity of a gene

**DOI:** 10.1186/1756-8935-4-8

**Published:** 2011-05-07

**Authors:** Vinesh Vinayachandran, Rama-Haritha Pusarla, Purnima Bhargava

**Affiliations:** 1 Centre for Cellular & Molecular Biology (Council of Scientific and Industrial Research) Uppal Road, Hyderabad-500007, India

## Correction

After publication of this work [1] we noticed that there was on inadvertent oversight due to which Figure 1 (Figure 5) and 2 (Figure 6) Legends were interchanged. While Figures and their captions are correct, Legends should have been interchanged and read as follows.

**Figure 5:**
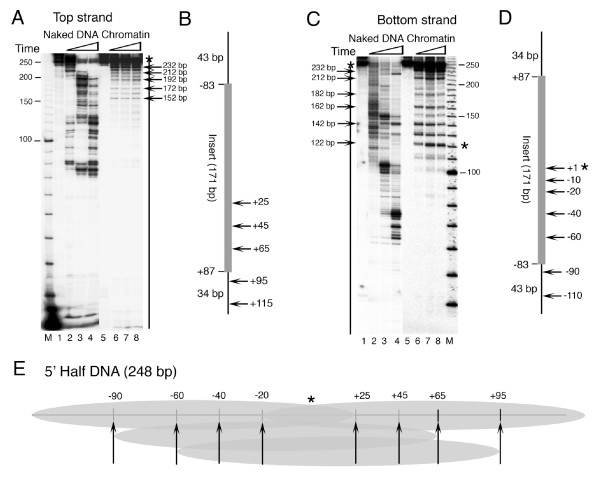
**Exo III mapping of the nucleosomes on the 5' Half *SNR6 *DNA fragments**. Chromatin was assembled on the 248 bp DNA fragments labeled at the 5' end of either of the strands and digested by Exo III for different times. A 10 bp DNA ladder (Invitrogen) was end labeled and used as size marker in the lanes M. (A) Mapping on the top strand. Lanes 1 and 5 show uncut DNA; lanes 2-4 show naked DNA and 6-8 show chromatin. Sizes of the marker bands are given in the left hand side while arrows on the right hand side give the sizes of the DNA fragments due to the pauses of Exo III. (B) Schematic summary of mapping results from the panel A. Insert represents the cloned genomic DNA from -83 to +87 bp positions in the *SNR6 *gene while the flanking 43 and 34 bp are vector-derived DNA. The numbers on right hand site are the Exo III stops seen in the gel and represent the nucleosome boundaries. (C) Mapping on the bottom strand. Lanes 1 and 5 show uncut DNA; lanes 2-4 show naked DNA and 6-8 show chromatin. Sizes of the marker bands are given in the right hand side while arrows on the left hand side give the sizes of the DNA fragments due to the pauses of Exo III. (D) Schematic summary, similar to panel B, of the mapping results from the panel C. The numbers on right hand site are the Exo III stops seen in the gels and represent the nucleosome boundaries. (E) Schematic representation of mapping results on 5' half DNA from both the strands. Ovals represent nucleosomes while arrows show positions of their boundaries and Exo III stops, marked in upper portion of the cartoon. An asterisk in panels C, D and E shows the position of Exo III stop in the middle of the DNA fragment, at +2 bp position of *SNR6*.

**Figure 6:**
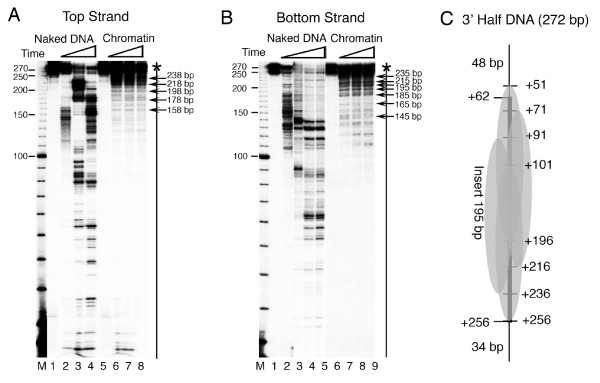
**Exo III mapping of the nucleosomes on the 3' Half *SNR6 *DNA fragments**. Chromatin was assembled on the 272 bp DNA fragments labeled at the 5' end of either the top strand (panel A) or the bottom strand (panel B). A 10 bp DNA ladder (Invitrogen) was end labeled and used as size marker in the lanes M. Sizes of the marker bands are given in the left hand side of both the panels while arrows on the right hand side give the sizes of the DNA fragments due to the pauses of Exo III. Lanes 1 and 5 in both the panels show the uncut DNA. Rest of the lanes show naked DNA or chromatin digested for different times by Exo III. (C) Schematic representation of mapping results from the panels A and B. Insert represents the cloned genomic DNA from +62 to +256 bp positions in the *SNR6 *gene while the flanking 48 and 34 bp are vector-derived DNA. The numbers on right hand site are the Exo III pauses seen in the gels and represent the nucleosome boundaries.
